# Deletion of the Actin-Associated Tropomyosin *Tpm3* Leads to Reduced Cell Complexity in Cultured Hippocampal Neurons—New Insights into the Role of the C-Terminal Region of Tpm3.1

**DOI:** 10.3390/cells10030715

**Published:** 2021-03-23

**Authors:** Tamara Tomanić, Claire Martin, Holly Stefen, Esmeralda Parić, Peter Gunning, Thomas Fath

**Affiliations:** 1 Dementia Research Centre, Department of Biomedical Sciences, Faculty of Medicine, Health and Human Sciences, Macquarie University, Sydney, NSW 2052, Australia; tamara.tomanic@hdr.mq.edu.au (T.T.); holly.stefen@mq.edu.au (H.S.); esmeralda.paric@mq.edu.au (E.P.); 2 Cellular and Genetic Medicine Unit, School of Medical Sciences, University of New South Wales, Sydney, NSW 2052, Australia; martin.claire@gmail.com (C.M.); p.gunning@unsw.edu.au (P.G.)

**Keywords:** neurite growth, actin cytoskeleton, protein segregation

## Abstract

Tropomyosins (Tpms) have been described as master regulators of actin, with *Tpm3* products shown to be involved in early developmental processes, and the *Tpm3* isoform Tpm3.1 controlling changes in the size of neuronal growth cones and neurite growth. Here, we used primary mouse hippocampal neurons of C57/Bl6 wild type and Bl6^Tpm3flox^ transgenic mice to carry out morphometric analyses in response to the absence of *Tpm3* products, as well as to investigate the effect of C-terminal truncation on the ability of Tpm3.1 to modulate neuronal morphogenesis. We found that the knock-out of *Tpm3* leads to decreased neurite length and complexity, and that the deletion of two amino acid residues at the C-terminus of Tpm3.1 leads to more detrimental changes in neurite morphology than the deletion of six amino acid residues. We also found that Tpm3.1 that lacks the 6 C-terminal amino acid residues does not associate with stress fibres, does not segregate to the tips of neurites, and does not impact the amount of the filamentous actin pool at the axonal growth cones, as opposed to Tpm3.1, which lacks the two C-terminal amino acid residues. Our study provides further insight into the role of both *Tpm3* products and the C-terminus of Tpm3.1, and it forms the basis for future studies that aim to identify the molecular mechanisms underlying Tpm3.1 targeting to different subcellular compartments.

## 1. Introduction

Tropomyosin (Tpm) is an actin-associated protein and key regulator of actin filament structure and dynamics in both muscle and nonmuscle cells. There are over 40 Tpm isoforms in mammalian cells expressed from four different genes: *Tpm1*, *Tpm2*, *Tpm3* and *Tpm4*. Out of these, *Tpm1*, *Tpm3* and *Tpm4* genes are active in neuronal cells. Expression of Tpm isoforms is spatially and temporally regulated and different isoforms segregate to actin filaments that are localized in different compartments within the same cell. *Tpm3* gene isoforms are the most studied nonmuscle cell Tpm isoform, of which Tpm3.1 has been the most well characterized.

*Tpm3* isoforms are generated via alternative splicing of the N-terminal exons 1a and 1b, the internal exons 6a and 6b and the C-terminal exons 9a, 9c and 9d. All *Tpm3* isoforms in neurons use the N-terminal exon 1b, resulting in the generation of low molecular weight *Tpm3* isoforms. Tpm3.1 isoform contains 6a internal and 9d C-terminal exons. *Tpm3* products localize to specialized subcellular compartments in neurons, including the growth cone of extending neurites [1,2,3], the postsynaptic compartment of central nervous system neuron synapses [4,5] and the axon initial segment [6]. Functional studies show that the overexpression of Tpm3.1 results in recruitment of myosin IIb to different actin filament populations [1], as well as the competition of exogenous Tpm3.1 with cofilin for actin filament binding sites in B35 cells [1]. Furthermore, exogenous Tpm3.1 localizes to filopodia and growth cones in mouse primary cortical neurons, which consequently display significantly enlarged growth cones and an increase in the number of axonal and dendritic branching [2].

Although exon 9d-containing isoforms are found at higher levels in the developing mouse brain compared to exon 9a- and 9c-containing isoforms, and the expression of exon 9d-containing isoforms decreases in older mice [5,7,8], it is important to highlight the demonstrated compensatory nature of *Tpm3* gene isoforms by which other *Tpm3* isoforms are upregulated in response to the knock-out (KO) of exon 9d-containing isoforms [3]. Consequently, the KO of Tpm3.1 leads to only subtle morphological changes in neurons, with a reduction in dendritic shaft length and growth cone size, as well as an increase in the number of primary branches of both axons and dendrites when compared to wild type neurons [3]. Compensation of *Tpm3* isoforms has also been shown for exon 9a- [8] and exon 9c-containing isoforms [9] and has been reviewed in detail in Curthoys et al. [10]. Isoform compensation is likely to explain the relatively mild phenotypes in functional studies, using primary neurons from mouse *Tpm3* knockout models. However, the complete loss of low molecular weight *Tpm3* isoforms by KO of exon 1b is embryonically lethal [9], highlighting the importance of *Tpm3* isoform expression for early development. The embryonic lethality of the *Tpm3* exon 1b KO mice has previously limited functional studies that aim to determine whether the overall expression level of *Tpm3* isoforms is critical for neuronal function.

In contrast to overexpression experiments, it is difficult to address the function of any *Tpm3* gene isoform simply by reducing its expression due to the previously stated compensatory mechanisms of alternatively spliced products, as well as the fact that conventional KO of *Tpm3* is detrimental to embryonic development. One of the most recent studies investigated the functional impact of Tpm3.1 in the axon initial segment (AIS) by the means of pharmacological inhibition and genetic targeting (Tpm3.1-specific shRNA and a conditional *Tpm3* KO mouse model (with a floxed exon 1b: B6-Tpm3^tm2(Δ1b(flox))Hrd^, referred to here as Bl6^Tpm3flox^)) and concluded that Tpm3.1 is required for the maintenance of the AIS [6]. The conditional KO mouse model described in this study (Bl6^Tpm3flox^) is the most promising model to meaningfully address the deletion of *Tpm3* products so far, as it allows for the timed deletion of all low molecular weight *Tpm3* products.

Here, we tested the functional effect of deleting all LMW products of the *Tpm3* gene using the above-mentioned model combined with Cre-mediated homologous recombination in cultured mouse hippocampal neurons. As Tpm is structurally a head-to-tail coiled-coil polymer that cooperatively binds to actin filaments [11], with the N- and C-terminus being essential functional domains and the source of isoform specificity [12,13,14], we hypothesized that deleting a portion of these termini will have significant impact on Tpm3.1 isoform functions and behaviours—most notably Tpm3.1 localization and segregation, and its impact on cell morphology. In this study, we show that the deletion of six amino acids at the C-terminus of Tpm3.1 has an impact on localization and segregation of Tpm3.1 to the tips of neurites, while the deletion of two amino acids at the C-terminus of Tpm3.1 still localizes to the tips but decreases the amount of F-actin pool at the growth cone. We also discuss the differential impacts of C-terminal Tpm3.1 deletion constructs on morphological aspects of neurons when compared to the expression of a full-length Tpm3.1 construct.

## 2. Materials and Methods

### 2.1. Culture and Transfection of NIH3T3 Cells

NIH3T3 cells were grown in high-glucose Dulbecco’s Modified Eagle Medium (DMEM, Life Technologies, Berkeley, CA, USA) containing 10% fetal bovine serum (10% FBS) and L-glutamine at 37 °C and 5% CO_2_, as described previously [15]. For experiments, cells were plated in either 6-well plates or chamber slides for transfection. For the purpose of RNA extraction, the cells were plated on 6-well plates at a density of 1 × 10^5^ cell/well and left overnight to attach. Transfection mixture (FuGENE 6, Roche, Basel, Switzerland) was prepared by adding 3 µL of transfection reagent to 100 µL of serum-free media and incubated for 5 min at room temperature. After adding 2 µg of DNA (hTpm3.1, hTpm3.1Δ2AA or hTpm3.1Δ6AA) and mixing, the mixture was incubated for 45 min at room temperature and then added dropwise to the cells. The cells were placed in the incubator for 24 h and then either harvested for Western blot analysis and/or cDNA synthesis, or fixed for immunocytochemical analysis.

### 2.2. Plasmids

The hTpm3.1 full-length construct was cloned and characterized previously [1]. hTpm3.1 deletion constructs were generated by amplification from the hTpm3.1 full-length construct, using the forward primer 5′–CAT GTC GAC ATG GCT GGG ATC ACC ACC–3′ for both hTpm3.1Δ2AA and hTpm3.1Δ6AA (containing a SalI restriction site), and the reverse primers 5′–GAC AAG CTT CTA ATT CAG GTC AAG CAG GGT–3′ (deleting the last two residues of the hTpm3.1 sequence and introducing a new stop codon and a HindIII restriction site downstream of the stop codon) and 5′–GAC AAG CCT CTA CTG GTC CAG CAT CCT TTG–3′ (deleting the last six residues of hTpm3.1 and introducing a new stop codon and HindIII restriction site) for hTpm3.1Δ2AA and hTpm3.1Δ6AA, respectively. Both hTpm3.1Δ2AA and hTpm3.1Δ6AA DNA fragments were first cloned into pGEM-T Easy vector (Promega, Wisconsin, USA) and then subcloned into pHβAPr-4(sig-) expression system [16] using SalI (BioLabs, Gloucester, MA, USA) at the 5′ end and HindIII (BioLabs, Gloucester, MA, USA) at the 3′ end.

### 2.3. Transgenic Mice Used in the Study

The Bl6^Tpm3flox^ line was designed with LoxP sites, flanking exon 1b of *Tpm3*, and was generated as previously described in [6]. All procedures involving animals were approved by Macquarie University Animal Care and Ethics Committee and conducted in accordance with national and international guidelines.

### 2.4. Primary Culture of Mouse Hippocampal Neurons

**Figure 1:**
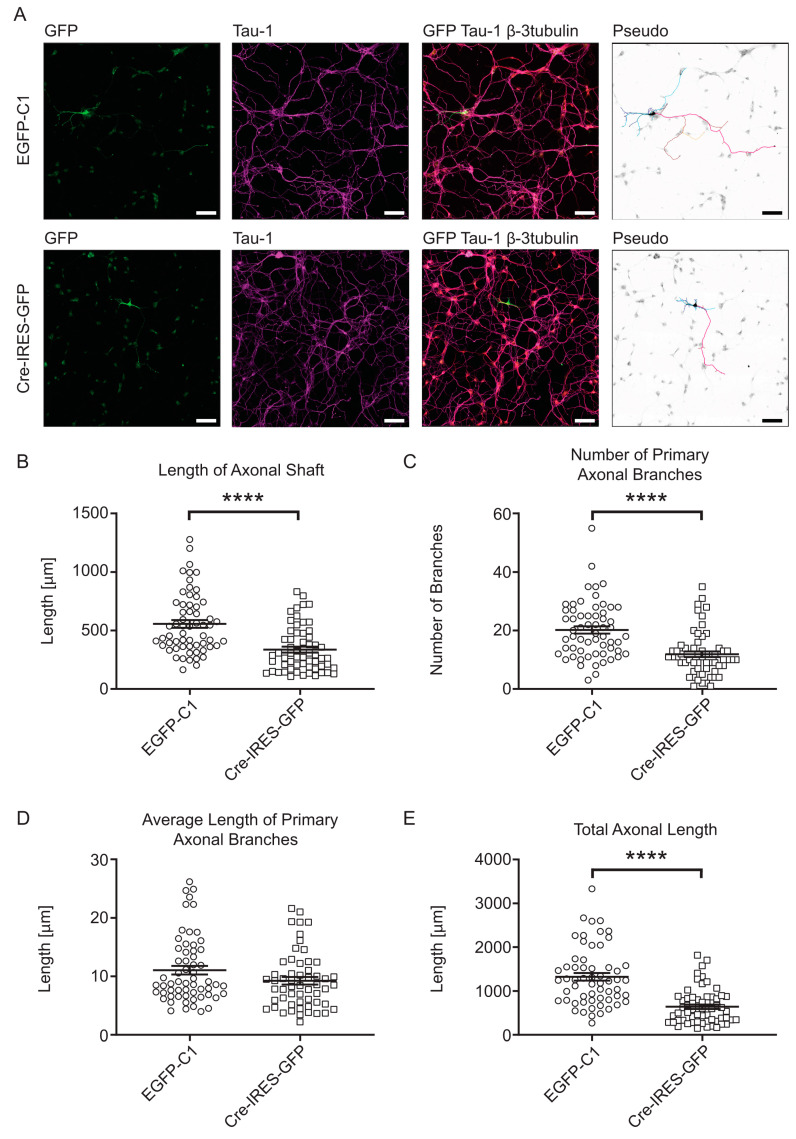
Effects of *Tpm3* knock-out on axonal morphology of Bl6^Tpm3flox^ neurons. Representative fluorescent images of mouse primary hippocampal neurons expressing EGFP-C1 and Cre-IRES-GFP for 96 hours, fixed at 4DIV and stained with GFP, Tau-1 and β3-tubulin antibodies. Pseudo images are inverted black and white GFP fluorescence images with pseudocoloured axon and dendrite labelling for clearer visualizations of the compartments. Curves in pink—axonal shaft, burgundy—primary axonal branches, orange—secondary axonal branches, light blue—dendritic shaft, dark blue—primary dendritic branches, and purple—secondary dendritic branches (**A**). Quantitative analysis of axonal morphology at 4DIV after transfection with EGFP-C1 and Cre-IRES-GFP constructs. The analysed axonal morphological parameters are (**B**) length of axonal shaft, (**C**) number of primary axonal branches, (**D**) average length of primary axonal branches, and (**E**) total axonal length. In total, 55–60 neurons from three biological replicates (with at least 15 neurons per replicate) were analysed for each construct. Error bars represent standard error of the mean. Significance was determined by Mann–Whitney nonparametric test: **** *p* < 0.0001. Scale bars = 50 µm.

**Figure 2:**
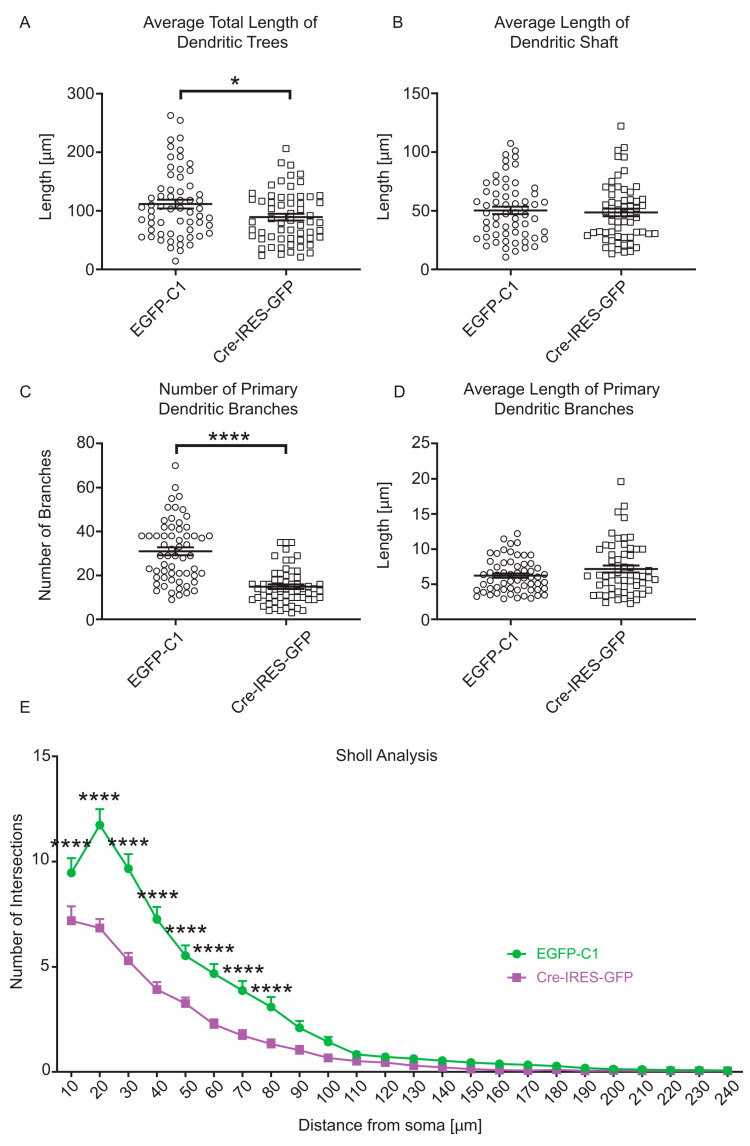
Quantitative analysis of dendritic morphology of Bl6^Tpm3flox^ neurons. Mouse primary hippocampal neurons expressed EGFP-C1 and Cre-IRES-GFP constructs for 96 h and were fixed at 4DIV. The analysed dendritic morphological parameters are: (**A**) average total length of dendritic trees, (**B**) average length of dendritic shaft, (**C**) number of primary dendritic branches, (**D**) average length of primary dendritic branches, and (**E**) Sholl analysis. In total, 55–60 neurons from three biological replicates (with at least 15 neurons per replicate) were analysed for each construct. Error bars represent standard error of the mean. Significance was determined by Mann–Whitney nonparametric test: * *p* < 0.05, and **** *p* < 0.0001. Significance of Sholl analysis was determined by nonparametric two-way ANOVA test with Sidak’s test for multiple comparisons: * *p* < 0.05, **** *p* < 0.0001.

Cultures of primary hippocampal cells were prepared from embryonal day 16.5 (E16.5) of C57/Bl6 mice used in the overexpression experiments, and Bl6^Tpm3flox^ mice used in KO experiments (Figure 1 and Figure 2). Mouse primary hippocampal neurons were prepared as described previously [17]. In brief, after removing the brains from E16.5 mouse embryos and isolating hippocampi with microscissors, the dissected hippocampi were placed in 2 mL of HBSS. This was followed by the addition of 250 µL of Trypsin (Sigma-Aldrich, Darmstadt, Germany) and incubation at 37 °C for 20 min. Next, 250 µL of Deoxyribonuclease I (DNase I, Sigma-Aldrich, Darmstadt, Germany) was added for 30 s followed by the addition of 10 mL DMEM (Life Technologies, Berkeley, CA, USA) containing 10% fetal bovine serum (DMEM/10% FBS, Hyclone, Utah, USA). Upon settling of the tissue to the bottom of the tube, supernatant was removed and replaced with fresh 10 mL DMEM/10% FBS for a thorough wash of the tissue and removal of the DNaseI. The tissue was triturated in 1 mL DMEM/10% FBS using fire-polished Pasteur pipettes. Upon trituration, the cells were plated on a poly-D-Lysine-coated 12 mm round glass coverslips at a density of 70,000 cells per well in a 24-well plate and incubated at 37 °C and 5% CO_2_ for 2 h in DMEM/10% FBS culture medium. After incubation, the medium was replaced with 1 mL per well of complete Neurobasal medium (NB/B27: Neurobasal, Life Technologies; supplemented with 2% B27, Life Technologies + 0.25% GlutaMAX, Invitrogen).

### 2.5. Transfection of Mouse Primary Hippocampal Neurons

Neurons were transfected with the following plasmids: pEGFP-C1 (Clontech, Mountain View, CA, USA), Cre-IRES-GFP (Addgene #48201), hTpm3.1 [1], hTpm3.1Δ2AA, and hTpm3.1Δ6AA. For this, 50% of culture media volume was collected from the wells of a plate that cells were cultured in (conditioned medium) and kept at 37 °C, 5% CO_2_. Cells were transfected using Lipofectamine 3000 (Thermo Fisher Scientific, Waltham, MA, USA) according to manufacturer’s protocols. Following the transfection, the cells were incubated at 37 °C and 5% CO_2_ for 90 min. After incubation, the media was aspirated, and conditioned medium was added to the cells. For Bl6^Tpm3flox^ primary hippocampal neurons, transfection procedure with Cre-IRES-GFP and EGFP-C1 plasmids was performed at 0DIV during cell plating. For C57/Bl6 primary hippocampal neurons, the transfection with EGFP-C1, hTpm3.1 hTpm3.1Δ2AA and hTpm3.1Δ6AA was performed at 2DIV.

## 3. Immunocytochemistry

Mouse primary hippocampal neurons and NIH3T3 cells were fixed in 4% paraformaldehyde for 15 min at room temperature and permeabilized in 0.1% Triton-X 100 (Sigma-Aldrich, Darmstadt, Germany) for 5 and 15 min, respectively. Blocking was performed in 2% FBS made in PBS for 30 min at room temperature and it was followed by incubation with primary antibodies at 4 °C overnight. After washing five times in PBS, the cells were incubated with secondary antibody for 30 min at room temperature, washed five time in PBS and then mounted in either Fluorsave (Sigma-Aldrich, Darmstadt, Germany) reagent or Prolong Gold antifade reagent with DAPI (Life Technologies, Berkeley, CA, USA) onto glass slides (Universal). Phalloidin staining was performed after secondary antibody washes, for 20 min at room temperature followed by washing in PBS three times. While phalloidin was diluted in PBS, all primary and secondary antibodies were diluted in 2% FBS and they include the following: mouse LC-1, (1:200; detects the epitope formed by first 10 amino acid residues from N-terminus of hTpm3.1, characterized by Schevzov et al. (2011) [13], and was a kind gift from Jim Lessard, Cincinatti Children’s Hospital Medical Center, Cincinnati, OH, USA), sheep γ-actin (1:1000; Schevzov et al. [18]), rabbit anti-GFP (1:500; Abcam ab290), mouse anti-MAP2 (1:500; Sigma-Aldrich M4403), chicken anti-β3-tubulin (1:250; Milipore ab9354), rabbit anti-MAP2 (1:500; Sigma-Aldrich M3696), mouse anti-Tau-1 (1:500; Milipore mab3420), anti-mouse Alexa-488, (1:1000; Molecular probes), donkey anti-sheep Cy3 (1:1000; Jackson ImmunoResearch Laboratories), donkey anti-rabbit Alexa-488 (1:500; Life Technologies A32790), donkey anti-mouse Alexa-555 (1:500; Life Technologies A31570), goat anti-chicken Alexa-647 (1:500; Life Technologies, A21449), donkey anti-mouse Alexa-488 (1:500; Life Technologies A32766), donkey anti-rabbit Alexa-555 (1:500; Life Technologies A31572), donkey anti-mouse Alexa-647 (1:500; Life Technologies, A31571), donkey anti-rabbit Alexa-647 (1:500; Life Technologies A31573), and 555-phalloidin (1:100; Life Technologies, A34055).

## 4. Imaging

NIH3T3 cells were imaged using a Jenoptik ProgRes CF Scan digital camera with ProgRes V2.5 software mounted on an Olympus BX50 microscope. The images were captured through U-MWV/U-MWIBA3/U-MWIY filters for DAPI/Alexa-488/γ-actin, respectively, using 40x objective lens and merged using Photoshop for 2 biological replicates, with 60–80 cells examined per replicate per experimental group. Mouse primary hippocampal neurons were imaged using Achroplan 40x/1.3 oil objective on a Zeiss Axioskop 40 fluorescent microscope system with the Axiocam 506 mono high-resolution camera and the ZEN2 lite software from Zeiss. At least 15–20 transfected neurons per coverslip from four coverslips were imaged per experimental group per biological replicate, which makes it 60–80 imaged neurons per biological replicate in total for each group.

Exposure times were kept consistent to make viability of fluorescence comparisons. The cells with very high expression levels were not chosen for analysis to prevent artefacts resulting from overexpression. The images were collected below the saturation level of the camera. All images shown are representative of results across all experiments.

## 5. Morphological and Statistical Analysis

To quantify the association of exogenous Tpms with actin filament stress fibres, the image analysis was performed using Coloc2 plugin in ImageJ software (v.2.1.0). Pearson’s correlation coefficient (PCC) was measured in one selected region of interest per transfected cell where stress fibres were clearly visible. The range of the area measured was between 10 and 60 μm^2^, with a consistent mean area between experimental groups (Supplemental Figure S1). PCC was determined between the 555 nm (γ-actin) channel and 488 nm (Tpm) channel. For each construct, between 60 and 80 cells, taken from 2 independent experiments, were analysed for each experimental group.

For C57/Bl6 cells, the dendritic compartment was identified as the MAP2/β3-tubulin positive compartment, while the axonal region was verified as the MAP2 negative/β3-tubulin positive compartment. For Bl6^Tpm3flox^ cells, the dendritic compartment was identified as Tau-1 negative/β3-tubulin positive, and axonal as Tau-1/β3-tubulin positive compartment. All images were processed in ImageJ (v. 2.1.0). For each experimental group, 15–20 neurons (~5 from each coverslip) were chosen from the 60–80 imaged neurons per biological replicate by using Random Number Generator (RNG). The analysis of processed images was carried out using Neurolucida software (MBF Bioscience, USA, v.2019.1.1), using the semiautomated approach; we first initiated the automatic tracing in AutoNeuron workflow to outline the somatic, axonal and dendritic compartments, after which we performed manual corrections and labelling of generated areas. After Shaft Ordering in Neurolucida, we used the Neurolucida Explorer package to conduct branched structure analysis and centrifugal Sholl analysis.

To perform the measurement of fluorescence intensity, data were collected manually by using segmented line or polygon selections tracing options in ImageJ to trace the 10% of total neurite length at both proximal and distal neurite ends (in 488 nm channel), or to outline the axonal growth cones (in 488 nm channel), respectively. In the case of growth cones, the 488 nm channel was overexpressed on purpose, to get a better outline of the edges, while fluorescence intensity was measured in the phalloidin (555 nm) channel. For segregation experiments, 13 neurons per experimental group (per biological replicate) were chosen via RNG to perform this analysis for the total of 3 biological replicates (39 per experimental group in total). For F-actin pool measurement, 20 neurons per experimental group (per biological replicate) were analysed for the total of four biological replicates (80 neurons per experimental group in total).

**Figure 3:**
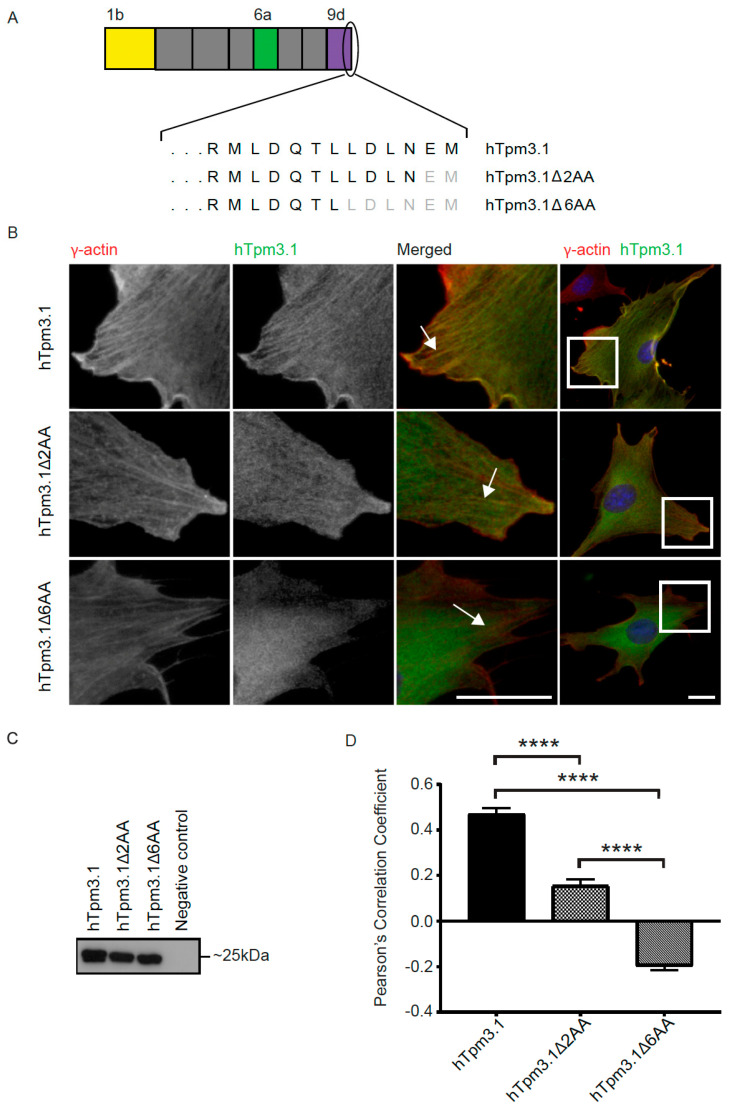
Expression of C-terminal deletion plasmids in NIH3T3 cells. NIH3T3 cells were transfected with hTpm3.1, hTpm3.1Δ2AA and hTpm3.1Δ6AA plasmids and fixed after 24 h. (**A**) Alternatively, spliced exons 1b, 6a and 9d that give rise to the hTpm3.1 isoform are shown in yellow, green and purple, respectively. Amino acids deleted at the C-terminus are given in grey for hTpm3.1Δ2AA and hTpm3.1Δ6AA. (**B**) NIH3T3 cells were stained with γ-actin and LC-1 (the areas with prominent stress fibres are highlighted). (**C**) Expression of hTpm3.1, hTpm3.1Δ2AA and hTpm3.1Δ6AA on Western blot. (**D**) Association between actin and hTpm3.1 was quantified via Pearson’s correlation coefficient (PCC). Significance was determined by Kruskal–Wallis test with Dunn’s correction for multiple comparisons: **** *p* < 0.0001. Scale bars are 5 µm.

Individual groups used for comparisons did not pass the tests for Gaussian distribution (three independent tests were performed: D’Agostino and Pearson normality test, Shapiro–Wilk normality test and Kolmogorov–Smirnov normality test); therefore, the comparison between the groups was carried out with nonparametric tests. The significance was determined in GraphPad prism software (version 9.0.0) by performing the following: Kruskal–Wallis test with Dunn’s corrections for multiple comparisons (Figures 3, 5–7 and 9, Supplemental Figures S1, S4 and S6), two-way ANOVA with Sidak’s test for multiple comparisons for Sholl analysis (Figures 2 and 6), and Mann–Whitney nonparametric test (Figure 1 and Figure 2 and Supplemental Figures S2 and S3). All statistical data are presented in detail in Supplemental Figure S7.

## 6. Western Blotting

Low bisacrylamide gels (12.5%) were prepared and allowed to set. Resolving gel recipe: 2.5 mL of 30% Acrylamide-Low Bis, 2.25 mL of 1 M Tris pH 8.8, 1.14 mL of water, 60 µL of 10% SDS, 2.4 µL of TEMED, 45 µL of 10% APS. Stacking gel recipe: 266 µL of 30% Acrylamide Bis, 250 µL of 1 M Tris pH 6.8, 1.45 mL of water, 20 µL of 10% SDS, 2 µL of TEMED, 15 µL of 10% APS. Protein samples (10 µg per well) were run on the gels at 100 V–120 V. The gels were transferred to PVDF membranes using BioRad Trans-Blot Turbo Transfer System at constant 25 V, 1 Amp for 45 min. Membranes were blocked in 5% skim milk made in 1xTBS-T for 1 h at room temperature. Blocked membranes were incubated with primary antibody (mouse LC-1, 1:1000) for 1.5 h at room temperature and washed 3 times in 1xTBS-T for 10 min. Next, membranes were incubated with secondary antibody (donkey anti-mouse HRP, 1:5000; Jackson ImmunoResearch Laboratories) for 1 h at room temperature and washed 3 times in 1xTBS-T for 10 min. The membranes were incubated for 5 min in a 1 mL mixture of luminol and oxidizing ECL reagent, and the film was exposed to the membranes for the appropriate amount of time before being developed with an X-ray processor.

## 7. Results

### 7.1. Knock-Out of Tpm3 Gene Shows Decreased Length and Complexity of Both Axons and Dendrites of Primary Mouse Hippocampal Neurons

To investigate morphological effects of the absence of *Tpm3* products, we used primary hippocampal neurons, derived from Bl6^Tpm3flox^ mice, allowing the KO of all exon 1b containing *Tpm3* isoforms through Cre-mediated homologous recombination (namely, Tpm3.1, Tpm3.2, Tpm3.3, Tpm3.4, Tpm3.5, Tpm3.7, Tpm3.8, and Tpm3.9 isoforms). Cre-mediated depletion of Tpm3.1 in hippocampal neurons derived from the same Bl6^Tpm3flox^ mouse line has been previously confirmed by Abouellez et al. (2020) [6]. To induce the KO of floxed 1b exon in Tpm3.1, we transfected hippocampal neurons at 0 day in vitro (DIV) with a Cre-IRES-GFP plasmid to induce deletion of the floxed *Tpm3* exon 1b. After fixation at 4 DIV, we performed immunocytochemistry by triple staining for GFP to enhance the GFP signal, for tau to determine the axonal compartment, and for neuronal β3-tubulin to outline the entire morphology of neurons in the culture (including the somato-dendritic compartment) (Figure 1A). Following fluorescence imaging, we carried out a morphometric analysis to characterize changes in axonal and dendritic growth (Figure 1B–E and Figure 2A–E).

Neurons expressing Cre-IRES-GFP showed significantly reduced axonal shaft lengths when compared to control neurons (Figure 1B). These axonal shafts contained significantly fewer sites from which primary axonal branches emerged (Figure 1C). Decreased axonal complexity is also evident by a reduction in secondary axonal branches when compared to control neurons (Supplemental Figure S2A). However, the average length of primary axonal branches was not changed in neurons expressing Cre-IRES-GFP when compared to control construct (Figure 1D). The total length of primary branches was still significantly lower in neurons expressing Cre-IRES-GFP than in control neurons (Supplemental Figure S2B). The decreased length of axonal shaft and primary axonal branches overall contributed to significantly reduced total axon length in Bl6^Tpm3flox^ neurons expressing Cre-IRES-GFP when compared to control neurons (Figure 1E).

Dendrites of Cre-IRES-GFP-expressing neurons showed a significant decrease in the average total length of dendritic trees (Figure 2A). However, the average length of the dendritic shafts did not change (Figure 2B) when compared to control neurons. The average total length of dendritic trees appears to be a direct consequence of the lower number of primary dendritic branches in Cre-IRES-GFP-expressing neurons (Figure 2C), rather than a consequence of differences in average length of primary dendritic branches which was not changed when compared to control neurons (Figure 2D). This decrease in complexity of dendrites is further confirmed by a significantly lower average number of primary branches per dendritic tree in Cre-IRES-GFP-expressing neurons (Supplemental Figure S3A), which overall contributed to a decrease in total dendritic length of these neurons when compared to control neurons (Supplemental Figure S3B). After performing Sholl analysis to further investigate the changes in dendritic complexity, we found that the most significant decrease in complexity occurred within a 10–80 µm distance from the soma in neurons expressing Cre-IRES-GFP when compared to control neurons (Figure 2E). Moreover, the number of dendritic trees is also significantly lower in Cre-IRES-GFP-expressing neurons, as well as the total length of primary dendritic branches when compared to control neurons (Supplemental Figure S3C,D).

### 7.2. The C-Terminus of the Tpm3 Isoform Tpm3.1 is Important for Incorporation of Tropomyosin into Actin Filaments

We next asked whether the C-terminus of Tpm3.1 is critical for the localization of Tpm3.1 to actin filaments. To visualize the efficiency of Tpm3.1 to bind to actin filaments, we used NIH3T3 cells that contain prominent stress fibres, built of actin filament bundles, which Tpm3.1 has been shown to associate with [1]. To examine the reliance of Tpm3.1 on its C-terminus to associate with actin filaments, we generated two human Tpm3.1 (hTpm3.1) deletion constructs, with a deletion of two or six amino acid residues at the C-terminus (Figure 3A). To visualize the localization of exogenous hTpm3.1, transfected NIH3T3 cells were fixed and immunocytochemically stained, using LC-1 and an antibody against γ-actin. Microscopy analysis showed that the association of both hTpm3.1Δ2AA and hTpm3.1Δ6AA mutants with actin stress fibres was reduced when compared to the hTpm3.1 full-length construct (Figure 3B). The efficiency of expression of the hTpm3.1 full-length and deletion constructs in transfected NIH3T3 cells was confirmed by SDS-PAGE using an antibody (LC-1) specific for hTpm3.1 (not detecting mouse Tpm3.1) (Figure 3C). Colocalization analysis confirmed association of hTpm3.1 with F-actin (PCC 0.47 ± 0.03), and suggests a weak association of hTpm3.1Δ2AA with F-actin (PCC 0.16 ± 0.03) and no association of hTpm3.1Δ6AA (PCC −0.2 ± 0.02) with F-actin (Figure 3D).

### 7.3. C-Terminal Truncation of hTpm3.1 Impacts the Effect of hTpm3.1 Expression on Neuronal Morphology

**Figure 4:**
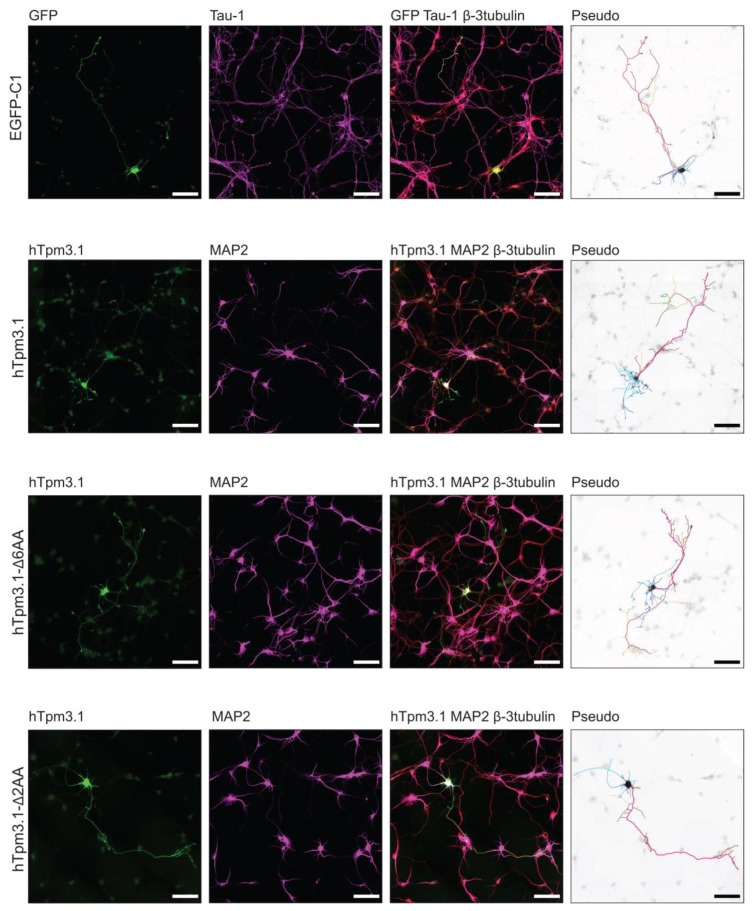
Effects of hTpm3.1 C-terminal truncation on morphology of C57/Bl6 hippocampal neurons. Representative fluorescent images of mouse primary hippocampal neurons expressing EGFP-C1, hTpm3.1, hTpm3.1Δ6AA and hTpm3.1Δ2AA for 48 h, fixed at 4DIV and stained with GFP, Tau-1 and β3-tubulin antibodies (EGFP-C1 transfected neurons) or LC-1, MAP2 and β3-tubulin (hTpm3.1, hTpm3.1Δ6AA, hTpm3.1Δ2AA transfected neurons). Pseudo images are inverted black and white GFP or LC-1 fluorescence images with pseudo-coloured axon and dendrite labelling for clearer visualizations of the compartments. Curves in pink—axonal shaft, burgundy—primary axonal branches, orange—secondary axonal branches, bright green—tertiary axonal branches, dark green—quaternary axonal branches, light blue—dendritic shaft, dark blue—primary dendritic branches, and purple—secondary dendritic branches. Scale bars = 50 µm.

To investigate the impact of C-terminal truncation of hTpm3.1 on neuronal morphology, the above described hTpm3.1Δ6AA- and hTpm3.1Δ2AA-expressing plasmids, as well as EGFP-C1- and hTpm3.1-expressing plasmids, were transfected into mouse primary hippocampal neurons at 2DIV and cultured for 4DIV in total. After fixation, the neurons were triple stained with GFP to enhance the GFP signal, with tau-1 to determine the axonal compartment and with β3-tubulin to outline the morphology of neurons (EGFP-C1 transfected neurons), or with LC-1 to identify neurons transfected with hTpm3.1, MAP2 to determine the somato-dendritic compartment, and β3-tubulin (hTpm3.1, hTpm3.1Δ6AA and hTpm3.1Δ2AA transfected neurons) (Figure 4). The morphometric analysis of fluorescent images, performed using Neurolucida software, showed significant differences between these four experimental groups of cultured neurons.

**Figure 5:**
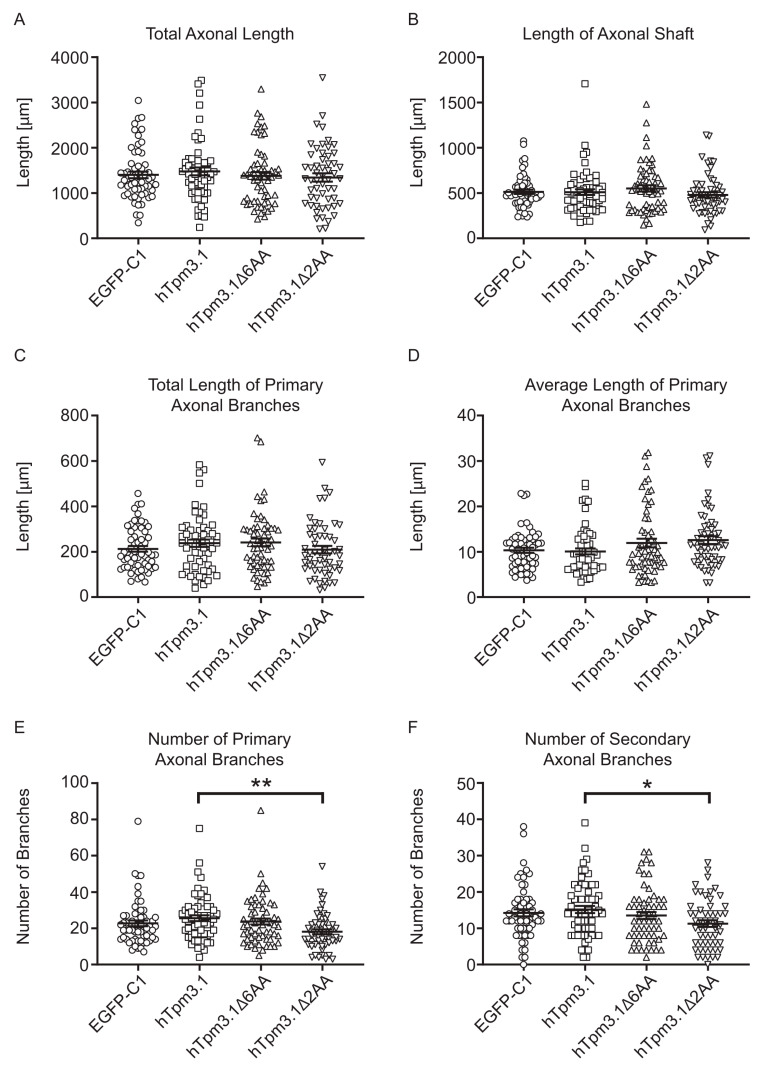
Quantitative analysis of axonal morphology of C57/Bl6 hippocampal neurons. Mouse primary hippocampal neurons expressed EGFP-C1, hTpm3.1, hTpm3.1Δ6AA and hTpm3.1Δ2AA for 48 h and were fixed at 4DIV. The analysed axonal morphological parameters are (**A**) total axonal length, (**B**) length of axonal shaft, (**C**) total length of primary axonal branches, (**D**) average length of primary axonal branches, (**E**) number of primary axonal branches, and (**F**) number of secondary axonal branches. In total, 55–60 neurons from three biological replicates (with at least 15 neurons per replicate) were analysed for each construct. Error bars represent standard error of the mean. Significance was determined by Kruskal–Wallis test with Dunn’s correction for multiple comparisons: * *p* < 0.05, ** *p* < 0.01.

Measurement and analysis of axons showed that there were no significant changes in the axonal length between different groups, which is reflected in the lack of changes in total axon length (Figure 5A), length of axonal shaft (Figure 5B), total length of primary axonal branches (Figure 5C), and average length of primary axonal branches (Figure 5D). However, hTpm3.1Δ2AA-expressing neurons showed a significant decrease in axonal complexity when compared to the expression of full-length hTpm3.1, which is demonstrated in the lower number of primary axonal branches (Figure 5E) and lower number of secondary axonal branches (Figure 5F). On the contrary, expression of mutant hTpm3.1Δ6AA did not display any difference in axonal complexity when compared to the hTpm3.1 full-length construct.

**Figure 6:**
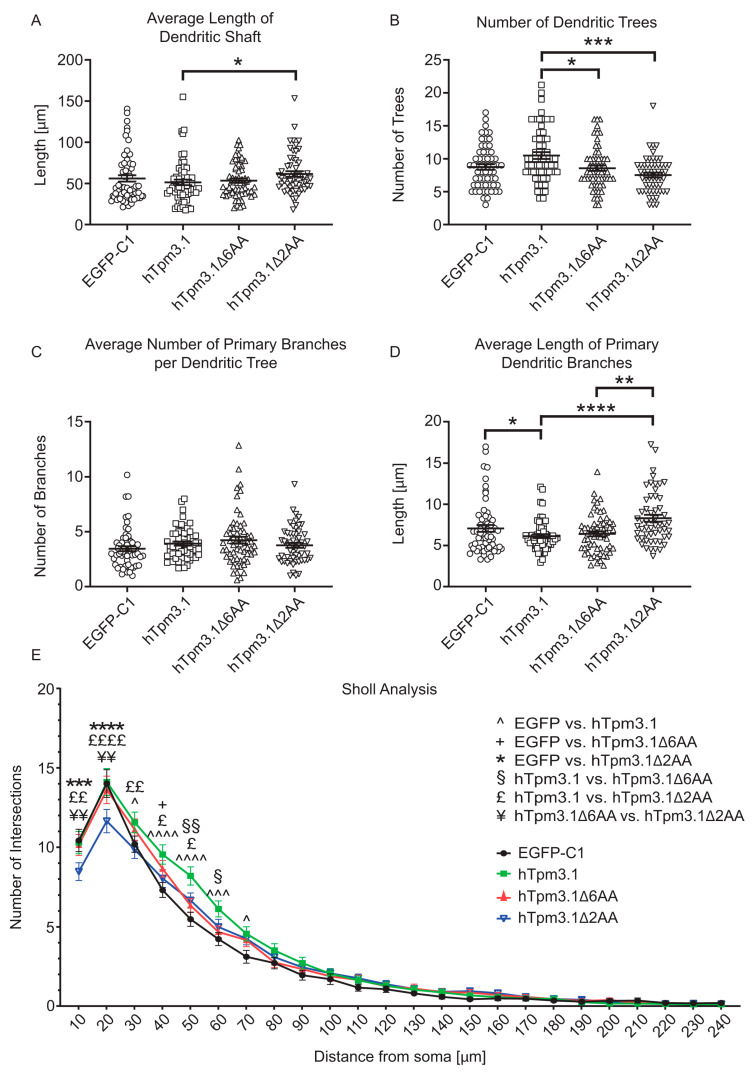
Quantitative analysis of dendritic morphology of C57/Bl6 hippocampal neurons. Mouse primary hippocampal neurons expressed EGFP-C1, hTpm3.1, hTpm3.1Δ6AA and hTpm3.1Δ2AA for 48 h and were fixed at 4DIV. The analysed dendritic morphological parameters are: (**A**) average length of dendritic shaft, (**B**) number of dendritic trees, (**C**) average number of primary branches per dendritic trees, (**D**) average length of primary dendritic branches, and (**E**) Sholl analysis. In total, 55–60 neurons from three biological replicates (with at least 15 neurons per replicate) were analysed for each construct. Error bars represent standard error of the mean. Significance was determined by Kruskal–Wallis test with Dunn’s correction for multiple comparisons: * *p* < 0.05, ** *p* < 0.01, *** *p* < 0.001, and **** *p* < 0.0001. Significance in Sholl analysis was determined by nonparametric two-way ANOVA test with Sidak’s test for multiple comparisons: * *p* < 0.05, ** *p* < 0.01, *** *p* < 0.001, and **** *p* < 0.0001. Significant difference between EGFP-C1 and other constructs is indicated by the following symbols: ^-hTpm3.1, + -hTpm3.1Δ6AA, and *-hTpm3.1Δ2AA. Significant difference between hTpm3.1 and other constructs is indicated by the following symbols: §-hTpm3.1Δ6AA and £-hTpm3.1Δ2AA. Significant difference between hTpm3.1Δ6AA and hTpm3.1Δ2AA is indicated by the symbol ¥.

Contrary to the absence of significant changes in axonal length, the analysis of dendrites showed that expression of mutant hTpm3.1Δ2AA leads to significantly increased average length of dendritic shafts when compared to the expression of full-length hTpm3.1 (Figure 6A).

However, the number of dendritic trees was significantly lower in mutant hTpm3.1Δ2AA- as well as hTpm3.1Δ6AA-expressing neurons when compared to the control (Figure 6B). Nevertheless, the dendritic trees showed no significant changes in their average total lengths between the groups (Supplemental Figure S4A). There were also no significant changes in the average number of primary branches per dendritic tree between constructs. However, the average length of primary dendritic branches was lowest in full-length hTpm3.1-expressing neurons, whilst neurons expressing mutant hTpm3.1Δ6AA and hTpm3.1Δ2AA showed significantly increased average length of primary branches when compared to the hTpm3.1 full-length construct (Figure 6D). While this parameter alone can falsely point to less complexity of neurons expressing full-length hTpm3.1, it conversely becomes clear that, when the number of dendritic trees is high, they would reciprocally contain primary branches of shorter average length, especially if the individual trees are more complex. All these dendritic traits considered, the higher overall dendritic complexity of full-length hTpm3.1-expressing neurons becomes evident as they show the significant increase in the total length of primary dendritic branches (Supplemental Figure S4B), as well as the total dendritic length (Supplemental Figure S4C) when compared to control EGFP-C1-expressing neurons. In these two dendritic parameters, however, neither hTpm3.1Δ6AA- nor hTpm3.1Δ2AA-expressing neurons showed any significant difference. Although the hTpm3.1Δ2AA mutant showed an increased average length of primary dendritic shaft as mentioned above (Figure 6A), the number of primary branches emerging from those shafts is significantly lower than in neurons expressing full-length hTpm3.1 (Supplemental Figure S4D). Expression of full-length hTpm3.1 also exhibits a higher number of primary dendritic branches when compared to control neurons. A Sholl analysis finally unravelled most of the observed differences in dendritic complexity between the four experimental groups of neurons (Figure 6E). When compared to the mutant hTpm3.1Δ2AA, expression of full-length hTpm3.1 resulted in an increased complexity within a 10–50 µm distance from soma, while the dendritic complexity increased more distal to the soma when compared to mutant hTpm3.1Δ6AA-expressing neurons. Expression of mutant hTpm3.1Δ6AA shows slightly increased dendritic complexity when compared to control neurons, only at the 40 µm distance from soma. Out of all the observed groups, expression of mutant hTpm3.1Δ2AA showed the lowest dendritic complexity, especially in close proximity to soma (10–20 µm). The significant difference between mutant hTpm3.1Δ2AA-expressing and both mutant hTpm3.1Δ6AA- and EGFP-C1-expressing neurons disappears at a 30 µm distance from the soma, while a significant difference in complexity between neurons expressing mutant hTpm3.1Δ2AA and full-length hTpm3.1 can still be observed up to 50 µm distance from soma.

### 7.4. C-Terminal Truncation of Tpm3.1 Impacts the Segregation of hTpm3.1 to the Tips of Neurites

**Figure 7:**
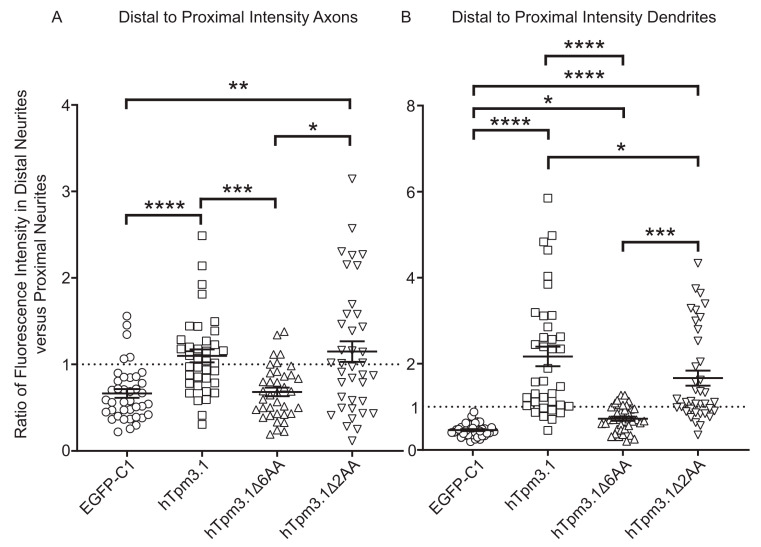
Effects of hTpm3.1 C-terminal truncation on segregation of hTpm3.1 to the tips of neurites. Mouse primary hippocampal neurons expressed EGFP-C1, hTpm3.1, hTpm3.1Δ6AA and hTpm3.1Δ2AA for 48 h and were fixed at 4DIV. Fluorescence intensity in the 488 nm channel (EGFP-C1 and LC-1) was measured as the average intensity along the 10% of the length of each neurite at either proximal or distal end. Difference of intensity at each end of neurite is displayed as a ratio of fluorescence intensity at distal end versus proximal end in (**A**) axons and (**B**) dendrites. 39 neurons from three biological replicates (with 13 neurons per replicate) were analysed for each construct. Error bars represent standard error of the mean. Significance was determined by Kruskal–Wallis test with Dunn’s correction for multiple comparisons: * *p* < 0.05, ** *p* < 0.01, *** *p* < 0.001, and **** *p* < 0.0001.

To explore the impact of C-terminal deletion of hTpm3.1 on its subcellular localization, the same neurons used for axonal and dendritic morphometry analysis were also screened for fluorescent intensity of the exogenously expressed protein in the proximal and distal regions of the neurites. Mean fluorescence intensity values along the most proximal and most distal 10% of neurite length were measured at 488 nm excitation for all experimental groups (Alexa488 secondary antibody labelling). The ratio of distal to proximal intensity was determined and compared between groups (Figure 7).

For both axons and dendrites, the ratio of fluorescence intensity in the distal to proximal region of neurites was above the value of 1 in neurons expressing full-length hTpm3.1 and mutant hTpm3.1Δ2AA, revealing that these two proteins segregate to the tips of neurites rather than close to soma (Figure 7A,B). This was not the case for the expressed EGFP-C1 control and mutant hTpm3.1Δ6AA, which were both more enriched closer to the soma (the fluorescence ratio was less than the value of 1). Furthermore, neurons expressing full-length hTpm3.1 and mutant hTpm3.1Δ2AA show a significantly higher difference in the ratio of fluorescence in the distal to proximal region of the neurite when compared to both EGFP-C1- and mutant hTpm3.1Δ6AA-expressing neurons. However, in dendrites there is a significant difference between full-length hTpm3.1- and mutant hTpm3.1Δ2AA-expressing neurons, where mutant hTpm3.1Δ2AA-expressing neurons exhibited less fluorescence intensity at the tips of dendrites than the full-length hTpm3.1-expressing neurons (Figure 7B).

### 7.5. C-Terminal Deletion of hTpm3.1 Significantly Impacts the Amount of F-Actin Pool at the Growth Cone

**Figure 8:**
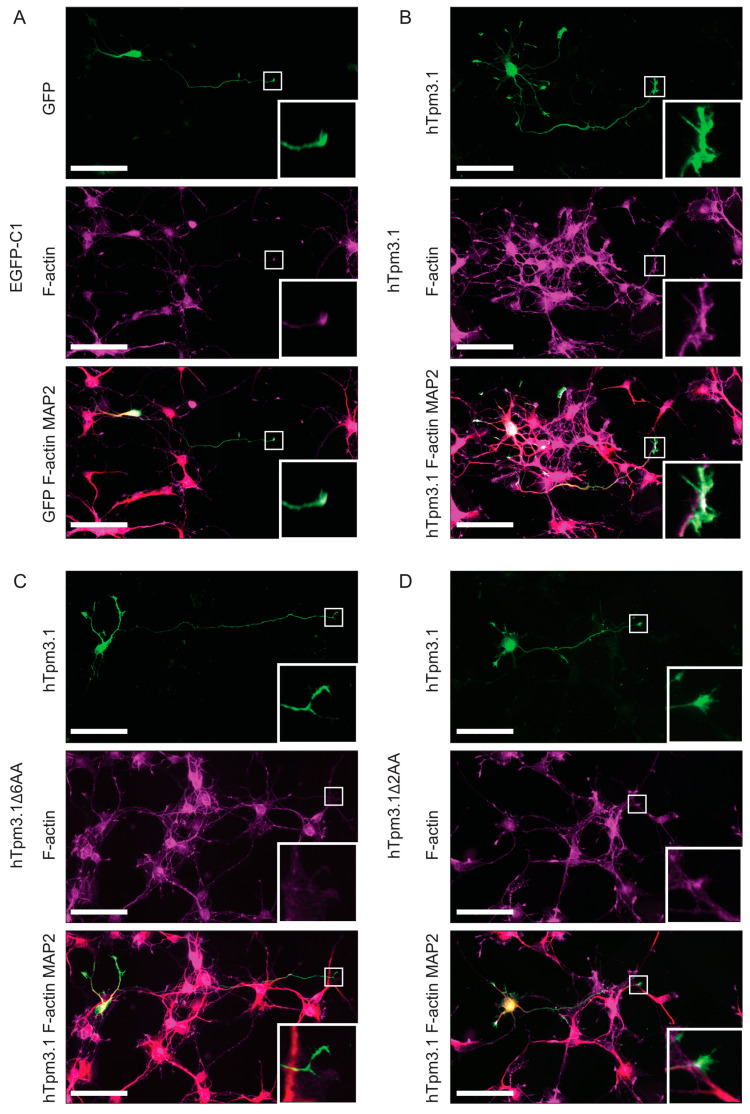
Effects of hTpm3.1 C-terminal truncation on the amount of F-actin pool in the growth cone of 3DIV old mouse primary hippocampal neurons. Representative fluorescent images of mouse primary hippocampal neurons expressing (**A**) EGFP-C1, (**B**) hTpm3.1, (**C**) hTpm3.1Δ6AA and (**D**) hTpm3.1Δ2AA for 24 h, fixed at 3DIV and stained with GFP, phalloidin and MAP2 antibodies (EGFP-C1 transfected neurons), or LC-1, phalloidin and MAP2 (hTpm3.1, hTpm3.1Δ6AA and hTpm3.1Δ2AA transfected neurons). Growth cones are highlighted and enlarged for each experimental group. The 488 nm channel was overexposed on purpose to obtain a better outline of the growth cone. Scale bars = 50 µm.

It was previously suggested that Tpms have regulatory roles in actin dynamics at growth cones [19]. Considering the above data (Figure 7), which propose a difference in segregation of C-terminally truncated Tpm3.1 to the neurite tips, we hypothesized that this will consequently impact the pool of F-actin at the growth cone. To investigate the differences in the amount of F-actin in the growth cone, mouse primary hippocampal neurons were transfected with EGFP-C1, full-length hTpm3.1, mutant hTpm3.1Δ6AA and mutant hTpm3.1Δ2AA at 2DIV, cultured for 3DIV in total, and triple stained with GFP to enhance the GFP signal, MAP2 to determine somato-dendritic compartment and phalloidin to visualize filamentous actin, or with LC-1 to detect hTpm3.1 transfected neurons, MAP2 and phalloidin (Figure 8).

**Figure 9:**
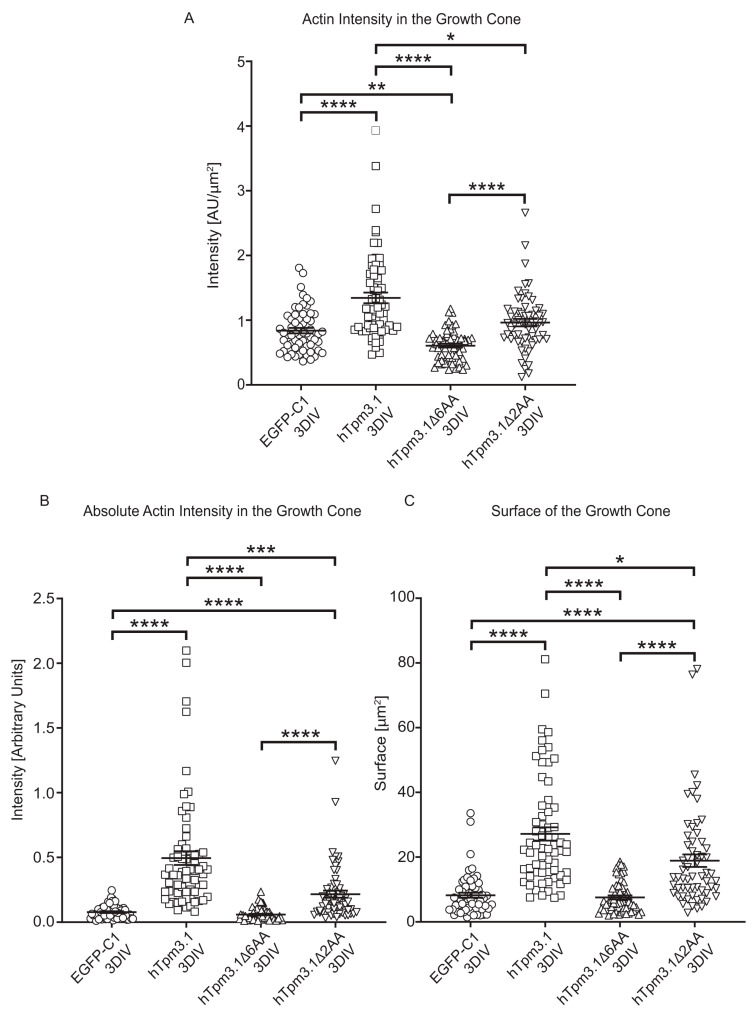
Effects of Tpm3.1 C-terminal truncation on the amount of F-actin at the growth cone. Actin intensity in the growth cone was measured as mean fluorescence intensity of the area of the growth cone in phalloidin channel (**A**). Absolute actin intensity in the growth cone was obtained by multiplying the value of actin intensity by the growth cone surface (**B**). Surface of the growth cone was measured by manually outlining the area of the growth cone and measuring the surface (**C**). 60–69 growth cones from four biological replicates (with at least 5 growth cones per replicate) were analysed for each construct. Error bars represent standard error of the mean. Significance was determined by Kruskal–Wallis test with Dunn’s correction for multiple comparisons: * *p* < 0.05, ** *p* < 0.01, *** *p* < 0.001, and **** *p* < 0.0001.

Neurons expressing hTpm3.1 and mutant hTpm3.1Δ2AA showed greater F-actin fluorescence intensity in the growth cone (Figure 9A), as well as greater absolute F-actin intensity (Figure 9B) and greater surface of the growth cone (Figure 9C) when compared to neurons expressing the hTpm3.1Δ6AA mutant construct. The full-length hTpm3.1 construct displayed significantly higher fluorescence intensity when compared to control neurons expressing the EGFP-C1 construct in both F-actin intensity and absolute F-actin intensity at the growth cone, and it also showed greater surface of the growth cone to that of control neurons. However, hTpm3.1Δ2AA-expressing neurons showed only significantly increased absolute F-actin fluorescence intensity at the growth cone when compared to control EGFP-C1 neurons, while there was no change in F-actin intensity. In addition, same significant difference profiles of F-actin pool in the growth cone have been observed in neurons cultured for 4DIV (Supplemental Figures S5 and S6), suggesting that the displayed behaviours between investigated groups are not the result of changes in development.

## 8. Discussion

The importance of *Tpm3* products in early development has been well documented so far [9] and due to previously mentioned compensatory mechanisms, it is difficult to investigate the isolated biological role of any endogenous *Tpm3* isoform. In this study, by using Bl6^Tpm3flox^ mouse primary hippocampal neurons, we showed the remarkable impact of the absence of *Tpm3* products on neuron morphology after just four days of *Tpm3* KO. Therefore, we confirmed Bl6^Tpm3flox^ as a mouse model useful in future research for timed and controlled *Tpm3* KOs at any required developmental stage. The significant decrease in all analysed morphological parameters, including axonal as well as dendritic length and complexity, suggests very rapid deterioration of neurons containing *Tpm3* KO. The biological significance of shorter and less complex neurites points to the decreased ability of a neuron to form potential synapses, to provide possible connections with neighbouring or distant neurons and glial cells, and to adequately respond to existing environmental cues. However, whether these synapses, connections or responses, even if in small numbers, would also be physiologically active remains to be investigated. This thought should also be kept in mind when considering the neuron morphological outcomes of C-terminal truncated mutants.

The N- and C-terminus have previously been shown to be the main source of the Tpm isoform specificity and their functional diversity [12,13,14]. However, the functional characterization of Tpms and their reliance on the integrity of the C-terminal end for the regulation of the actin cytoskeleton is very limited. This study is the first to identify the importance of the C-terminus of the major *Tpm3* isoform Tpm3.1 for its segregation to different subcellular compartments within neuronal cells. We also demonstrate that the regulation of the F-actin pool via Tpm3.1 is dependent on the integrity of the C-terminal end of Tpm3.1.

Previous studies have demonstrated that Tpms have a regulatory role in actin dynamics at neuronal growth cones as reviewed in detail by Schevzov et al. [19], and that exogenously expressed Tpm3.1 localizes to filopodia and growth cones in mouse primary cortical and hippocampal neurons, which leads to growth cone enlargement and an increase in the number of axonal and dendritic branching [2,3]. In this study, we confirmed the enlargement of growth cones as well as the increased number of dendritic branches with the overexpression of hTpm3.1; however, the use of C-terminal truncated mutants also allowed us to address the question whether the C-terminus of Tpm3.1 is required to drive the observed changes in neuronal morphology. Significantly enlarged growth cones as well as the increased fluorescence intensity of F-actin at the growth cones in neurons transfected with hTpm3.1 and hTpm3.1Δ2AA suggests that Tpm3.1 drives polymerization of actin in the growth cones of the neurons of these two experimental groups. As previously stated in Gomez et al. [20], the two main forces that establish growth cone motility are protrusive forces for exploration of filopodia and lamellipodia at the growth cone generated via polymerization of actin filaments and traction forces that pull the growth cone forward, generated via the interaction of actin filaments with myosin II motor protein. The contribution of Tpm3.1 to generation of both traction forces has been previously elucidated in [1], where it has been shown that the exogenous Tpm3.1 recruits myosin IIb to different actin filament populations, while its role in generation of protrusive forces (displayed in the same study) suggests that expression of Tpm3.1 correlates with the displacement of ADF severing protein from actin filaments. Moreover, it has been shown that some tropomyosin isoforms inhibit both cofilin severing and Arp2/3 branch formation [21,22]. In this study, we have confirmed the presence of hTpm3.1Δ2AA at the growth cone (Figure 7) and have also shown a significant decrease in actin intensity and growth cone surface compared to that of hTpm3.1 (Figure 9), which suggests that deletion of two amino acid residues at the C-terminus of hTpm3.1 does not impact localization of this isoform to different cell compartments, but that it does impact F-actin polymerization at the growth cone. Whether the two amino acid C-terminal truncation of Tpm3.1 also influences the function of tropomyosin relevant to actin filament severing or myosin IIb recruitment, as shown in previous studies, is yet to be elucidated.

Deletion of six amino acid residues at the hTpm3.1 C-terminus appears to have even more detrimental effect on F-actin polymerization at the growth cone to that of deletion of two amino acid residues (Figure 8 and Figure 9). We suggest that this is due to the deficiency of mutant hTpm3.1Δ6AA when associating with F-actin, as is clearly demonstrated in NIH3T3 cells (Figure 3) and hTpm3.1Δ6AA not localizing to neurite tips (Figure 7), rather than exhibiting a concrete function at the growth cone and consequently decreasing the amount of F-actin. This is confirmed by hTpm3.1Δ6AA displaying a similar segregation profile to that of a freely diffusing control EGFP-C1 control construct in neurons (Figure 7). The lack of mutant hTpm3.1Δ6AA segregating to the distal part of axons and dendrites may also result in the attenuated impact of mutant hTpm3.1Δ6AA expression on both dendritic and axonal growth parameters, relative to mutant hTpm3.1Δ2AA when compared to hTpm3.1 expression. Overall, the expression of mutant hTpm3.1Δ6AA has only minor effects on these neurite growth parameters compared to the expression of the EGFP-C1 control, which is likely due to its deficiency to associate with F-actin and segregate to the correct subcellular compartment in neurons (Figure 5 and Figure 6 and Supplemental Figure S4). Many potential Tpm isoform sorting mechanisms have been proposed so far [12], including: (1) differential affinities of Tpms to specific actin filaments [23,24], where the role of Tpm C-terminal ends was implicated in determining the actin affinity [25]; (2) the activity of formins initiating conformational modifications along the actin filaments, predisposing them to bind differential Tpm isoforms [26,27]; (3) post-translational modifications of Tpms by signalling molecules [28,29]; (4) the regulation of the chemical environment that holds differential pH or ionic concentrations (Ca^2+^ sensitive actin-binding proteins described in Fukushima et al. [30] and Sobue et al. [31]; (5) the local activity of Rho GTPases [32]. Our study shows the significance of the C-terminus of *Tpm3* isoforms in their subcellular sorting. It also shows that different manipulations to the C-terminus can lead to differential sorting outcomes. The exact mechanisms that are responsible for the C-terminus-dependent sorting of *Tpm3* isoforms will require further investigation. Nevertheless, it is worth noting that the N- and C-termini of adjacent Tpm monomers in a Tpm polymer overlap by 11–14 amino acid residues, and that the amino acid sequence of a Tpm molecule contains periodic repeats along F-actin which it associates with [11], suggesting that any manipulation of the Tpm termini might lead to altered cooperative binding to the actin filament. It is possible that the deletion of six amino acids, and not that of two amino acids at the C-terminus of hTpm3.1 was sufficient to significantly impact either the periodicity of the Tpm polymer and/or the ability of Tpm to maintain its affinity and cooperative binding to F-actin. This purely mechanistic consequence of C-terminal manipulation could also explain the observed morphological changes in mutant hTpm3.1Δ2AA-expressing neurons—i.e., a significant decrease in axonal and dendritic complexity, while, at the same time, a significant increase in the length of dendritic shaft when compared to hTpm3.1-expressing neurons. In addition, it can be speculated that deleting portions of the C-terminus might lead to changes in subcellular targeting due to alterations in mRNA sorting. It has been shown that the C-termini of many proteins are required for proper trafficking of mRNAs to different subcellular compartments for either ER-targeting or localized translation: C-terminal translational pausing site mediates recruitment of the Xbp1 mRNA-ribosome-nascent chain complex to the ER membrane in *Drosophila* [33], the C-terminal RGG box/RG-rich region is required for neuronal RNA granule formation in *Xenopus* [34], while mutations in the C-terminal domain of TDP-43 alter trafficking of its mRNA along cortical axons of mouse primary neurons [35].

Cells utilize a range of mechanisms of transport either at the mRNA or protein level. How the C-terminal truncation of Tpm3.1 impacts these mechanisms still remains to be elucidated. Although there are some examples of the C-terminal region in targeting mRNA, there is increasing evidence that the fate of mRNA localization is via its 3′-UTR. However, the plasmids used in this study do not express the 3′-UTR of Tpm3.1, while its proper localization is still happening. This leads to the conclusion that the C-terminus is involved in targeting of Tpm3.1 within the cell. It would be of great importance to dissect this targeting mechanism and whether it includes C-terminal targeting, vesicle transport, or some of the more recently characterized means of protein transport such growth cone-like waves in neurons, which have been shown to transport filamentous actin from the soma to the neurite tips [36].

## 9. Conclusions

Previous work has established the important role of the tropomyosin isoform Tpm3.1 in several neuronal functions. Data from our current study, using conditional *Tpm3* KO mice as well as C-terminal deletion constructs, provide further detail on the role of *Tpm3* isoforms in neurite growth and reveal the dependence of the major *Tpm3* isoform Tpm3.1 on the intact C-terminus for its subcellular segregation and association with F-actin. The relationship between the C-terminus-dependent segregation and the ability of Tpm3.1 to regulate growth cone function will be crucial to better understand how neurons control neurite growth during development and in regenerating neurites. Of particular interest will be to identify the molecular mechanisms such as the transport pathways that underlie the targeting of *Tpm3* isoforms to the tip of extending neurites.

## Data Availability

The individual data points included in the main and supplemental figures of this manuscript can be found at osf.io/ds6qn/, DOI 10.17605/OSF.IO/DS6QN. All other relevant data are provided within the manuscript.

## Supplementary Materials

The following are available online at https://www.mdpi.com/2073-4409/10/3/715/s1: Supplemental Figure S1. Comparison of surface areas used to measure the association between stress fibres and exogenous tropomyosin across experimental groups. Supplemental Figure S2. Quantitative analysis of axonal morphology of Bl6Tpm3flox neurons. Supplemental Figure S3. Quantitative analysis of dendritic morphology of Bl6Tpm3flox neurons. Supplemental Figure S4. Quantitative analysis of dendritic morphology of C57/Bl6 hippocampal neurons. Supplemental Figure S5. Effects of hTpm3.1 C-terminal truncation on the amount of F-actin pool in the growth cone of 4DIV old mouse primary hippocampal neurons. Supplemental Figure 6. Effects of hTpm3.1 C-terminal truncation on the amount of F-actin at the growth cone. Supplemental Figure S6. Effects of hTpm3.1 C-terminal truncation on the amount of F-actin at the growth cone.
