# Supplementary material for: Deletion of the Actin-Associated Tropomyosin Tpm3 Leads to Reduced Cell Complexity in Cultured Hippocampal Neurons—New Insights into the Role of the C-Terminal Region of Tpm3.1
Source: Cells. 2021 Mar 23;10(3):715. doi: 10.3390/cells10030715 (PMC8005004; doi:10.3390/cells10030715)
Supplement: Supplementary file 1 [file cells-10-00715-s001.pdf]

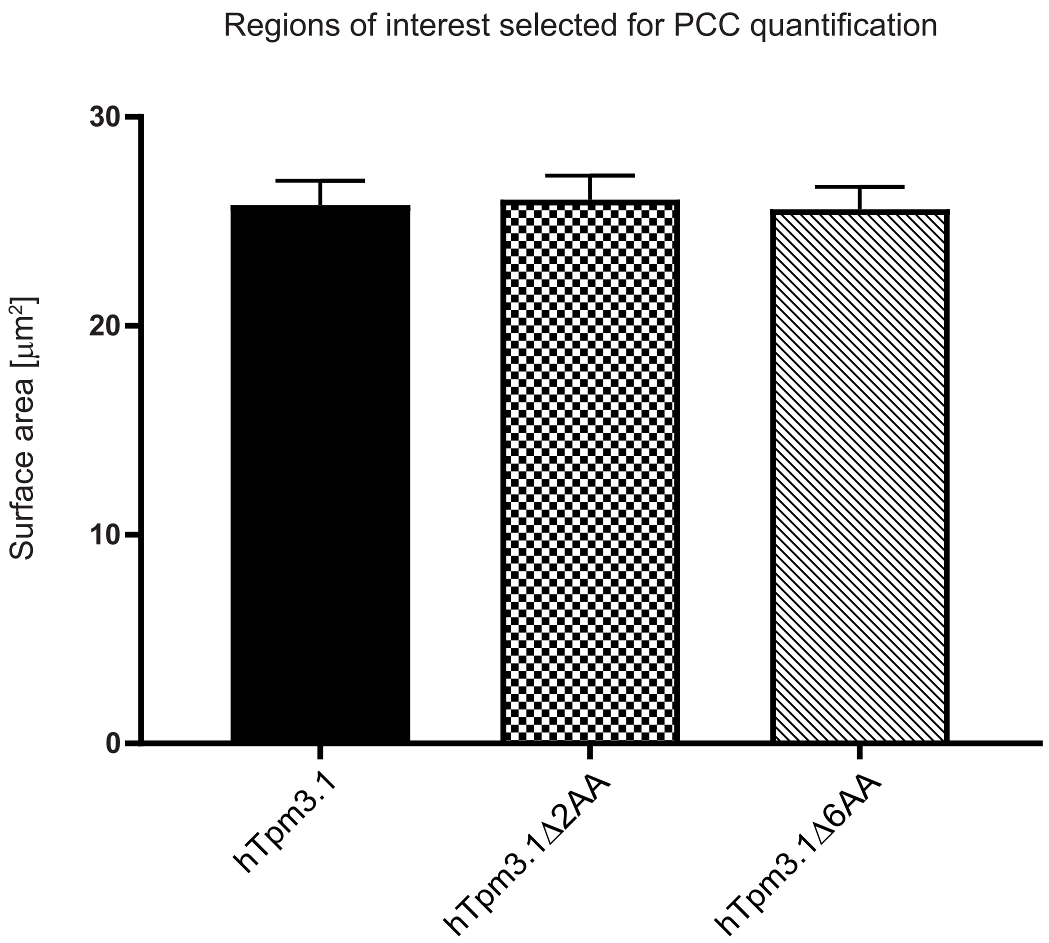

Supplemental Figure 1

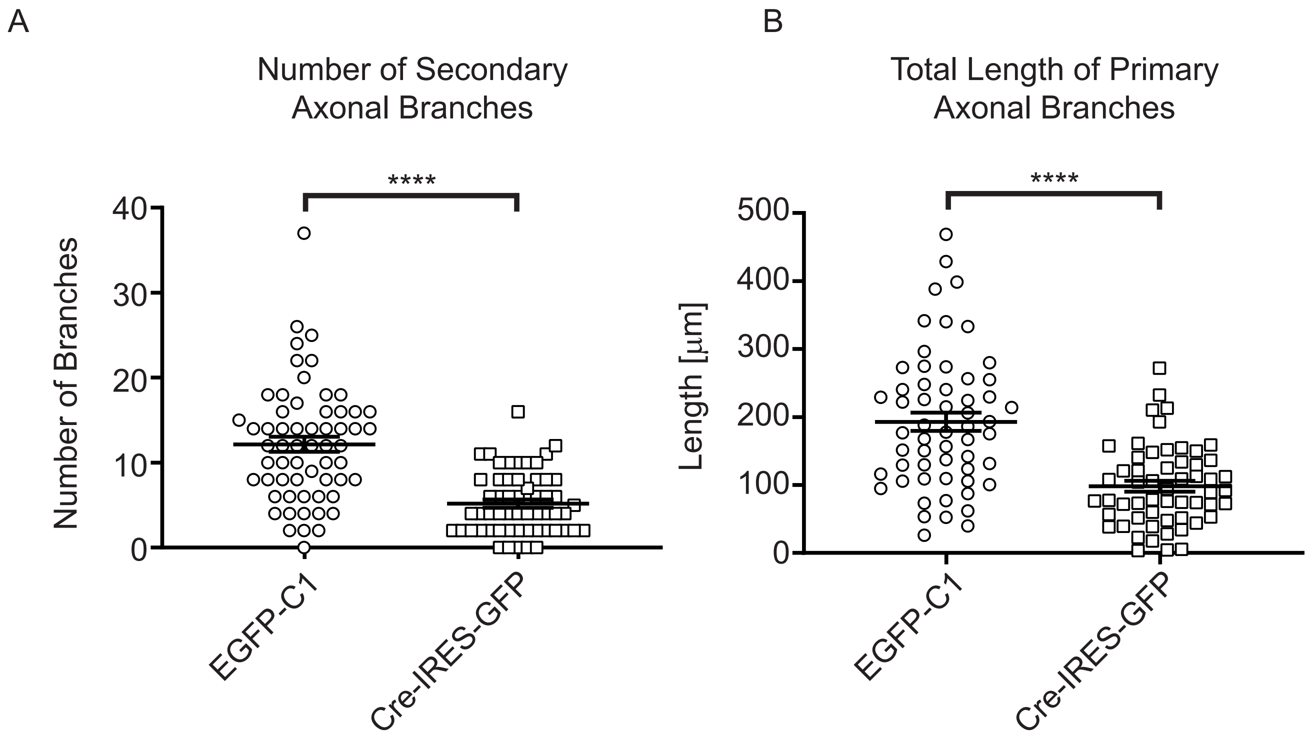

Supplemental Figure 2

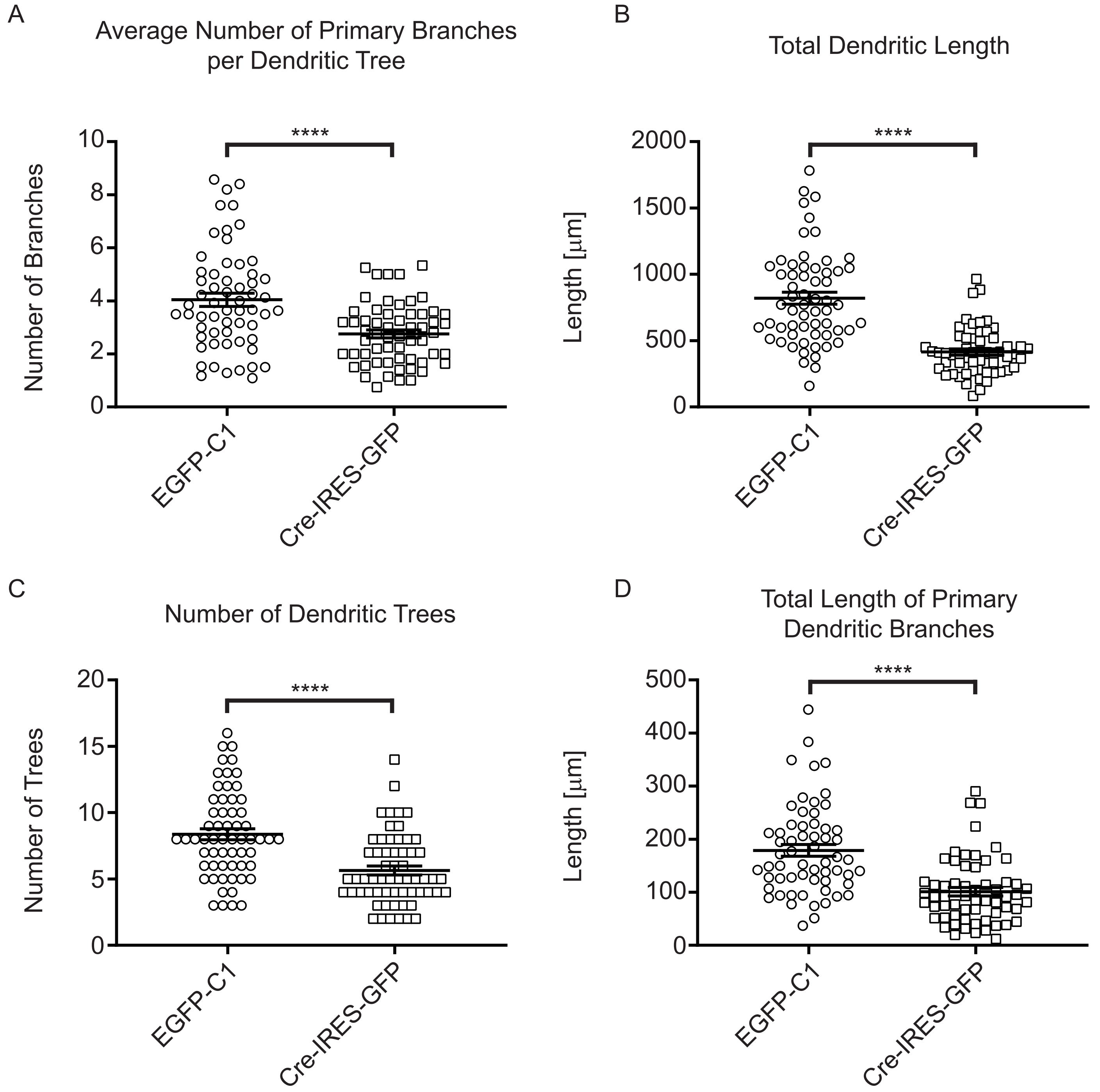

Supplemental Figure 3

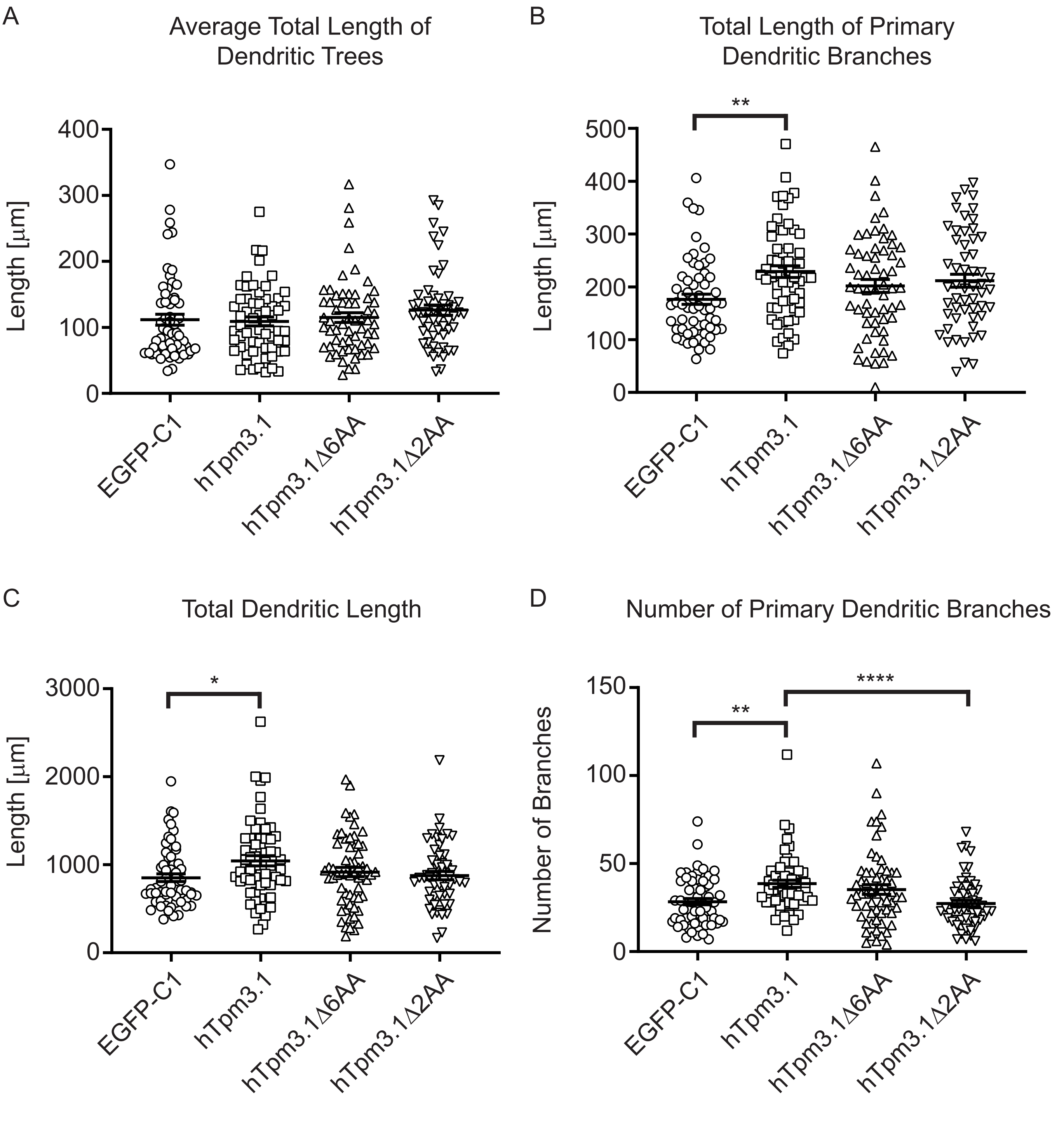

Supplemental Figure 4

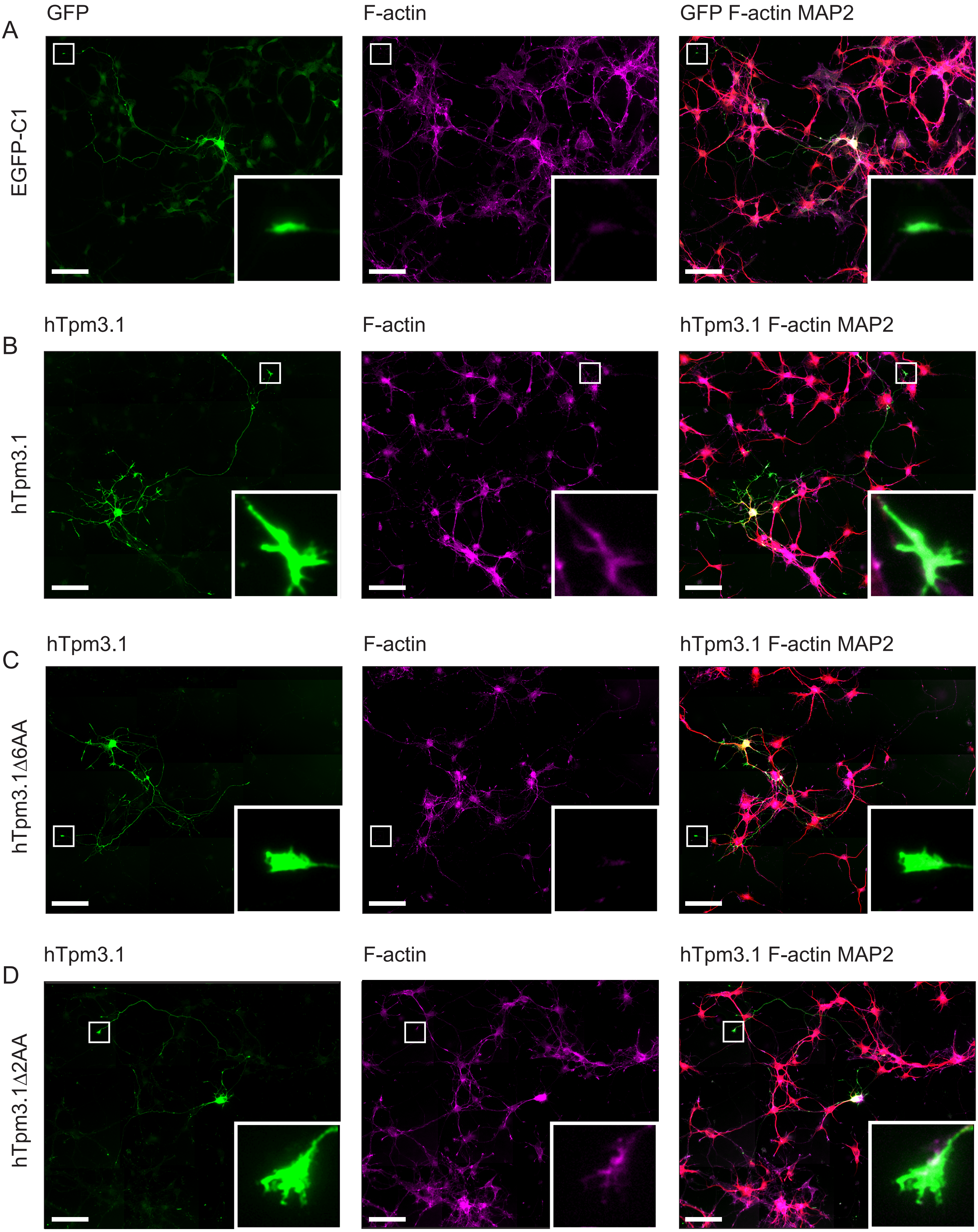

Supplemental Figure 5

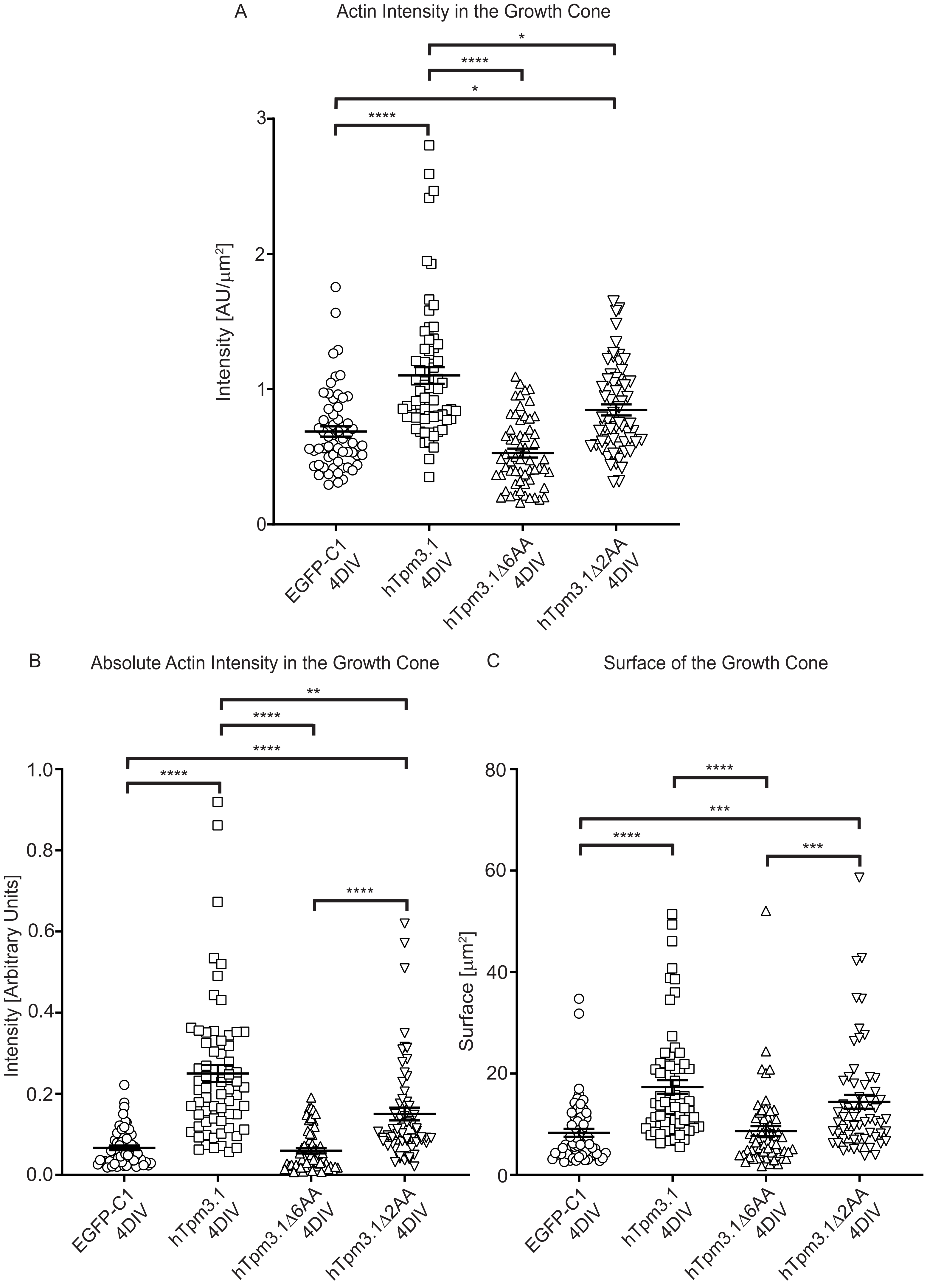

Supplemental Figure 6
